# Some Problems in Dental Histology, Physiology and Patho-Histology That Need to Be Solved*Read before the San Francisco District Dental Society, February 3, 1919.

**Published:** 1919-05

**Authors:** John S. Marshall

**Affiliations:** U.S.A.


### SOME PROBLEMS IN DENTAL HISTOLOGY, PHYSIOLOGY AND PATHO-HISTOLOGY THAT NEED TO BE SOLVED*

BY CAPT. JOHN S. MARSHALL, U.S.A., M.D., SC.D., F.A.C.S.

My object in preparing this paper is to encourage, if I can, a greater interest in the subject of *original investigation* and *research* in the field covered by the specialty of dental surgery, and to stimulate some of the young men already in the profession, and those contemplating entering it, to be willing to devote their time, their talents and their energies to the solving of some of the many problems—scientific problems—that yet remain, and are waiting to be satisfactorily solved.

Dental science, if I may say it, is not so far advanced as it should be with all the advantages that it has had for progress, and I believe the reason for this lies in the fact that we have all been too anxious to corral the elusive dollar. We all want to be rich; but I can assure you research work holds out no inducements along these lines. The research worker will always be poor, until some already rich people with more than they can spend are willing to give some of it to found dental research laboratories and provide for the maintenance of a certain number of young men with brains and enthusiasm who will devote their lives to this work.

It requires great courage and devotion upon the part of a young man to give his life and labors to science without a proper compensation. Fame and cheers do not feed and clothe wife and babies.

I started out with saying that there are many problems—scientific problems—that yet remain, and waiting

* Read before the San Francisco District Dental Society, February 3, 1919.

to be satisfactorily solved. May I enumerate a *few* of these unsolved problems?

*What do we really know about dental caries,* the most common of all diseases that afflict mankind? Our time, our talents, and our energies have been so fully occupied in trying to combat its progress and to repair the damages that it has wrought, that we have given little time and less attention, comparatively speaking, to seeking its cause and methods of prevention.

When I first began the study of dental pathology, the profession had settled upon the seeming fact that dental caries was caused from various acids taken into the mouth as food, condiments and medicines, or from acids rising from the stomach as products of imperfect digestion, or produced within the mouth from fermentation of food debris, as taught by Robertson, Westcott, Watts, Allport, and others.

A little later came the germ theory of the disease as taught by Leber and Rottenstein of Germany, of Mills and Underwood of England, and finally by Miller, who belonged to America, who claimed that the bacterium lactis was the principal cause of the disease.

At the present time wo do not fully subscribe to the views of Miller, for we are now taught that there are many other microorganisms in the mouth capable of forming acids by fermentation.

What agencies are responsible for susceptibility and immunity to dental caries? Why is one individual with a clean mouth distressingly susceptible to the ravages of the disease and another individual, showing a disgustinly filthy mouth and who is a complete stranger to the toothbrush, entirely immune?

What do we really know about pyorrhea alveolaris? Is it a constitutional or a local disease, or is it a local manifestation of a constitutional disease? Are the constitutional symptoms due to the local manifestations? Is it a disease of the gingivae or of the peridental membrane or of the alevolar process, or of all three combined? Is it caused by a constitutional virus? The presence of calcareous deposits, or the action of micro-organisms like the staphylococcus, the streptococcus, or the ameba-buccalis? Can it be cured by constitutional treatment alone, or by local treatment alone, or by both combined? Can it really be cured at all, except by the removal of the diseased teeth?

What do we really know about the physiological process of the eruption of the teeth, either deciduous or permanent? What are the forces which cause the teeth to move from their crowded and heretogeneous positions and assume their regular and normal alignment? Can anyone give a satisfactory answer to this question?

What do we know about the salivary secretions? What are the nerve forces which in one instance cause a flow of fluid of an alkaline reaction, in another a neutral, and in still another an acid reaction? Could we discover this and be able to control it at will, the two great problems of susceptibility and immunity would be solved.

What do we know about the enamel? Is it a vitrified substance having no vitality or nutrition, and nothing but a coat of mail? Or is it a vital substance (tissue) endowed with a certain amount of resistance to disease and possessing a degree of nutrition? What do we know of the cause which produce the denudation of the enamel? That mysterious wasting away of this tissue, until the labial half of the crown of the tooth, in exceptional cases, is entirely destroyed down to the gingival line and not infrequently exposing the pulp. This is one of the problems that most investigators have quietly side-stepped. Nevertheless, it is an important problem and worthy the steel of any Knight of Science. Our textbooks help us but little in the solving of these problems and often lead us astray by foolish or only partially digested theories.

Lord Bacon has said, "Books must follow science, not science books." He has also said, "Some books are to be tasted, others to be swallowed, and some few to be chewed and digested." I well remember as a young man listening to that long-departed nestor in dentistry, Dr. Wm. H. Atkinson, who, when speaking of the misleading statements to be found in many of our published books, would say in his most scathing manner, "Throw books to the dogs and study nature." Nature is an open book to those who can read her language. Wordsworth has said:

"Come forth into the light of things,
Let Nature be your teacher."

I would not, however, discourage the writing of books, for many times when one has a message to deliver it is the only way to get it before the profession. Our periodical literature would seem to be the place, the best place, for any message that we might desire to place before the profession. But you have seen, and so have I, dental journals three and four months old lying on the center-table of the waiting-room of a popular dentist with the mailing wrappers still on them. Not much for reading are we?

*What are the phenomena that cause secondary caries?* Most of you will say faulty manipulation. To some this means imperfect cavity preparation. To others it means faulty placing and consolidation of filling material. To still others, failure to observe the great principle laid down by Dr. G. V. Black of extension for prevention (extension to immune areas); while still others will contend that the cause lies in failure to restore proper approximal contact. But when all of these principles have been conscientiously observed, secondary caries will still accur. Why?

Doctors Flagg, Chase and Palmer, if living, would at once affirm that it was due to incompatibility of the filling material with the dental tissues. In other words, to electrical action between the tooth and the filling material, producing decomposition of the saliva and the formation of acids at the margin of the filling. Were they right or wrong? Has anyone as yet satisfactorily solved this problem? These questions can only be solved by laborious, painstaking investigation and research. I learned many years ago the truth of the saying, "Fools can ask questions that wise men can not answer."

The most of our methods of treatment are empirical. They do not rest upon a sound scientific basis and will not bear careful scientific analysis. We have fallen into the habit of following this, that, or some other method of treatment because some man, setting himself up as a teacher, has said his method was the only one that would bring about a successful cure. He may have been a clinical teacher, or a writer for the journals, or both, or an itinerant exploiter, and, because he has the convincing manner of speech and has been well advertised, we have swallowed his teaching without investigation and later have found, to our sorrow, many times, that we have been deceived. In a conversation with a traveling salesman, who had at one time been a practising dentist, and was at that time introducing a new drug, or rather a new application of an old and well-known drug, he remarked that he "found professional men, physicians and dentists the most gullible people and easy marks." "All a man needs is a good story and a glib tongue, and he can usually put it over."

You all remember how easily the physicians and dentists of the land were gulled by the good story and the glib tongues of the agents that presented that sure cure for pyorrhea alveolaris—ipecac—emetin, alcresta tablets, and the many other preparations of ipecac.

How many of us escaped the wiles of these artists?—for they were real artists in the presentation of the subject. Why are we so easily duped? Where lies the trouble? I admit the answer is beyond me, and so I, therefore, leave it up to you.

I am of the opinion, however, that if we would meet these gentlemen with the statement, "Friend, I am from Missouri," that it would have a wonderful chilling effect upon the story and the glibness of the speech. To the man that wants to know, and will know, the man who requires proof and not verbal statements, these gentlemen and their story, presented ever so glibly and convincingly, has very little effect.

The Good Book says, "Prove all things, hold fast that which is good." This is equally wise in science as in religion.

Huxley has said, "A little knowledge is dangerous, but where is the man that has so much of it as to be out of danger?"

Though "knowledge is power," as stated by Lord Bacon, this is not sufficient. Huxley, if I may quote him again, says, "The great need of life is not knowledge, but action." This last statement should be, to my mind, the motto of every young man. Knowledge, though of great value, is useless unless it is employed.

During the past two and a one-half years it has been my privilege and great good fortune to spend from three to five hours each day, except Sundays, in the private laboratory of Professor H. M. Evans, Chief of the Department of Anatomy of the University of California, studying the morphology and histology of the dental tissues. A portion of these studies has been confined to the human dental tissues, more particularly to the dentin and the enamel. The greater portion of my studies, however, have been associated with certain of the lower animals, the smaller mammals, and principally those that are generally designated as laboratory animals. These animals were selected for this purpose for three reasons. *First,* because dental morphology is so similar in all mammals as to make it unnecessary in every instance to employ human embryos; *Second,* because the laboratory animals are easily procured and readily studied; *Third,* because they are always under the immediate supervision and control of the research worker.

Although the problems which I have under investigation are intensely interesting from the standpoint of the student of dental histology, physiology and patho-histology, I am, nevertheless, constantly struck with wonder and admiration at the marvelous minuteness and exactitude with which the Supreme Architect has carried out the details of His creations, and like Kepler, the great astronomer, I am constrained to say "I thank God that I am permitted to think His thoughts over after Him."—*Pacific Dental Gazette.*

